# Removing unnecessary obstacles to randomised trials

**DOI:** 10.1186/1745-6215-14-S1-I2

**Published:** 2013-11-29

**Authors:** Rory Collins

**Affiliations:** 1 Clinical Trial Service Unit & Epidemiological Studies Unit (CTSU), Oxford University, Oxford, UK

## 

Since their introduction in the middle of the 20^th^ Century, randomised trials of sufficiently large size have provided reliable assessments of the safety and efficacy of treatments that have produced substantial improvements in health. During the past decade, however, increasingly onerous (albeit well-meaning) regulation and related bureaucracy have made clinical trials much more difficult and costly to conduct. The impact of these large increases in costs and effort is that many existing and novel interventions are not being evaluated, or the trials that are conducted are smaller and less informative than they might otherwise be. The negative effect of these obstacles on the emergence of reliable evidence about the safety and efficacy of treatments affects the care of people not just in developed countries but also in developing countries where resources are more limited and the burden of disease is large. Hence, there is an urgent need for major changes to procedures for the initiation, conduct, monitoring and safety reporting of clinical trials such that they are more proportionate to the risks of doing a trial compared with the risks of not doing it. Unless radical improvements are now made to the regulatory environment, the huge potential of clinical trials to assess the safety and efficacy of new and existing treatments and, thereby, to produce substantial improvements in health care and public health will not be fulfilled.

